# Exploring the Role of Artificial Intelligence in Mental Healthcare: Progress, Pitfalls, and Promises

**DOI:** 10.7759/cureus.44748

**Published:** 2023-09-05

**Authors:** Gemma Espejo, Wade Reiner, Michael Wenzinger

**Affiliations:** 1 Psychiatry and Behavioral Sciences, University of California, Irvine School of Medicine, Irvine, USA; 2 Psychiatry, University of Washington, Seattle, USA; 3 Psychiatry, Washington University in St. Louis, St. Louis, USA

**Keywords:** digital psychiatry, psychiatry, facilitators of innovation use in healthcare, healthcare innovation, artificial intelligence (ai), artificial intelligence in medicine, artificial intelligence in healthcare, mental health services

## Abstract

The rise of artificial intelligence (AI) heralds a significant revolution in healthcare, particularly in mental health. AI's potential spans diagnostic algorithms, data analysis from diverse sources, and real-time patient monitoring. It is essential for clinicians to remain informed about AI's progress and limitations. The inherent complexity of mental disorders, limited objective data, and retrospective studies pose challenges to the application of AI. Privacy concerns, bias, and the risk of AI replacing human care also loom. Regulatory oversight and physician involvement are needed for equitable AI implementation. AI integration and use in psychotherapy and other services are on the horizon. Patient trust, feasibility, clinical efficacy, and clinician acceptance are prerequisites. In the future, governing bodies must decide on AI ownership, governance, and integration approaches. While AI can enhance clinical decision-making and efficiency, it might also exacerbate moral dilemmas, autonomy loss, and issues regarding the scope of practice. Striking a balance between AI's strengths and limitations involves utilizing AI as a validated clinical supplement under medical supervision, necessitating active clinician involvement in AI research, ethics, and regulation. AI's trajectory must align with optimizing mental health treatment and upholding compassionate care.

## Editorial

Recent and rapid developments in artificial intelligence (AI) place us at the precipice of perhaps the biggest revolution in medical care to date. Already, applications and advances in AI can be found peppered across all levels of healthcare. A 2021 review of AI in mental healthcare covers many of these advances, which range from applying clinical algorithms and incorporating data from multiple electronic health record (EHR) systems to utilizing neuroimaging, genetic, and speech data to comment on prognosis for depressive disorders, future substance use, suicide risk, and functional outcomes. AI also allows for the acquisition of information outside of physician-patient encounters, utilizing information from smartphones or wearable devices, offering real-world, continuous data to aid the physician in decision-making and treatment [1]. Furthermore, innovation has already found a direct perch for AI in treatment through such applications as therapy for children with autism spectrum disorders, who have been found to react positively to robots who can help develop social skills [2]. While the advancements in AI applications continue to move forward at a dizzying pace, our ability to evaluate and critique limitations may struggle to keep up. More than ever, it is critical for clinicians to stay appraised of a field that not long ago may have seemed to have little relevance to our practice. The bright promises AI may bring toward solving inequalities and inefficiencies can also create stark shadows of over-mechanization or ersatz expertise.

As with all tools, users must be aware of pitfalls and limitations. Mental disorders are complicated and heterogeneous in nature, and any practicing psychiatrist can speak on the biopsychosocial model at play with all mental health issues. Disease states within psychiatry can rarely be tracked or diagnosed with objective numerical data, unlike other medical disorders. A systematic review from 2023 that focused on studies from 2016-2021 sheds light on significant limitations in AI mental health research. First, studies are largely retrospective without external validation and with a high risk for bias. This review also found that only 28% of studies used original data with over 70% of studies using information from databases or secondary analysis of clinical trials that were not designed for the purpose of AI-related study [3].

Additionally, the use of AI in healthcare raises concerns about the privacy of health information, including tracking and misuse of information by third parties. As mentioned, AI has the risk of bias and is currently not capable of self-reflection. Moreover, AI has the risk of further entrenching existing biases. Concerns about human overreliance on AI for future therapeutic interventions have also been raised, considering constant access that is not available to human clinicians [2]. Considering ongoing worries about “technology addiction” with video games and social media, an unhealthy relationship with AI may be something that patients and providers encounter in the future. Others have raised concerns about AI replacing rather than supplementing in-person healthcare, as well as the availability of AI services being used as an excuse to decrease in-person services, creating further disparities in access to health [2]. Governing bodies, such as the Food and Drug Administration, have recently made guidance statements on psychedelic research; a similar requirement for regulatory oversight for AI-related research and practice is another measure that would ensure quality and equity.

As we think about the future directions of AI, we must assume that AI will be applied in talk therapy. One can look to other industries to see this trend in action. Chatbots are utilized as the initial, affordable, always accessible, “low-touch-no-touch,” self-service option in a tiered approach to customer support while the “white glove” human support is left for customers with the highest needs or support tier. AI remains a potential option, as the marketplace of psychotherapy is ripe for disruption. While efficacious, quality psychotherapy is often inaccessible and expensive. Presumably, an AI therapist could provide a scalable, convenient, and affordable means to deliver basic teachings of cognitive reframing, validation, acceptance, thought defusion, and other psychological tools. For this to take place, an AI therapy service would likely have to create trust [2], offer feasibility, and demonstrate clinical efficacy, the latter of which is often not measured in studies [4]. Furthermore, clinicians may have to have further acceptance of AI utilization in clinical practice [1]. Perhaps physicians could play leadership or liaison roles in future AI-focused work groups, similar to how quality improvement groups have become standardized components of any healthcare system.

At an institutional level, healthcare systems have many decisions to make regarding stakeholders for design and implementation, governance, quality control, and long-term maintenance of AI-related tools such as clinical decision support (CDS). Already we can see a variety of approaches for who ‘owns’ AI integration. For instance, a survey of 34 health systems revealed a variety of organizational setups for deploying predictive models of AI: 50% utilized a decentralized translational approach that is driven primarily by research teams while 40% utilized an AI-healthcare team-driven approach that extends the native EHR configuration. Only 10% of surveyed systems utilized an IT-department-led approach, which relies on third-party model vendors and native EHR vendors [5]. This IT-department-led approach may become the dominant organizational setup due to more durability and use of off-the-shelf, scalable tools, with the downside of less novel model development. The growth in AI-CDS tools will likely heighten the importance of the centralized IT department. Moreover, there may be a growing role for physician informaticists who, through their dual understanding of technology and patient care, can help systems decide whether these technologies should be obtained through custom solutions versus off-the-shelf solutions and whether their implementation is likely to provide value to the health system, its clinicians, and its patients.

Importantly, there are unanswered questions regarding how AI tools will impact healthcare professionals. In an optimistic future, AI tools that ambiently listen to the interview will generate clinical notes, giving doctors more time to spend with their patients. CDS will aid physician decision-making, improving the quality of care. In a pessimistic future, AI tools will exacerbate already hot topics like moral injury, loss of autonomy, and scope of care. AI tools that increase efficiency may lead to ever-higher expectations for revenue generation and productivity. Algorithms could learn from physicians’ own documentation to facilitate the replacement of physicians by non-physicians. Perhaps doctors’ own documentation will be used to train AI models without physician input, consent, or remuneration. Similar issues are currently being litigated with artists whose work has been used without their consent to train generative, art AI systems such as Midjourney. It reasons that this issue would come to medicine sooner than later, and it has. In mid-August of 2023, in the same week, both Zoom and Simple Practice raised alarms when updates to their privacy led to widespread fear that the content of virtual visits would be data mined for AI tool development and corporate profit at the expense of patient privacy.

An ideal future is one in which AI provides a well-validated supplement to clinical care while remaining under the supervision and scrutiny of those with appropriate medical training so as to provide evidence-based, equitable care. As the technological revolution of AI races forward, we must react quickly to the benefits and pitfalls revealed in its path. This is needed to best support its optimization for treating mental health while minimizing its usurpation of the necessary human element for compassionate care. For these reasons, it is imperative for clinicians to take an active role in research, development, ethical commentary, and regulation of AI to best serve our patients.
